# A case report of gastric linitis plastica diagnosed by endoscopic ultrasound-guided fine needle aspiration

**DOI:** 10.1097/MD.0000000000008937

**Published:** 2017-12-15

**Authors:** Shinji Muraoka, Kouhei Tsuchida, Mari Iwasaki, Naoya Izawa, Hidehito Jinnai, Toshinori Komatsubara, Misako Tsunemi, Fumi Sakuma, Ken Kashima, Ko Fukushi, Hideyuki Hiraishi

**Affiliations:** Department of Gastroenterology, Dokkyo Medical University, Shimotsuga, Tochigi, Japan.

**Keywords:** esophagogastroduodenoscopy, fine needle aspiration, gastric fold swelling, gastric linitis plastica, signet ring cell carcinoma

## Abstract

**Rationale:**

There is currently no consensus on the ideal method for obtaining deep tissue biopsy material of advanced gastric LP. EUS-FNA has potential as a useful diagnostic method. Thus, we report the case of a 46-year-old male with advanced gastric linitis plastica (LP) who was diagnosed using endoscopic ultrasound-guided fine needle aspiration (EUS-FNA).

**Patient concerns:**

The patient underwent esophagogastroduodenoscopy (EGD) because of epigastric pain at a local clinic. The gastric fold swelling was pointed out by the EGD and despite the suspected advanced gastric LP, biopsy indicated Group 1. Repeat biopsy did not suggest malignancy. The patient was referred to our institution.

**Diagnoses:**

Endoscopic ultrasound indicated gastric wall thickening mainly in the greater curvature of the gastric corpus. Low-level echoes were detected throughout the entire gastric wall, and gastric wall layers had been disappeared. EUS-FNA of the gastric wall indicated signet ring cell carcinoma.

**Interventions:**

As a result of EUS - FNA, it became a policy to administer chemotherapy. In accordance with the patient's wishes, he was referred to another institution for chemotherapy.

**Outcomes:**

Normal biopsy did not give a definitive pathological diagnosis, and final diagnosis of LP was obtained with EUS-FNA.

**Lessons:**

We expect that EUS-FNA can be utilized as a relatively non-invasive, highly sensitive, and specific pathological diagnostic procedure for advanced gastric LP. EUS-FNA should be considered as one way to obtain a deep tissue biopsy of advanced gastric LP.

## 1 Introduction

Stage IV gastric cancer, sometimes referred to as advanced gastric linitis plastica (LP), is defined as the characteristic thickening and hardening of the gastric wall without marked ulceration or elevation and is distinguished by an undistinguishable unclear boundary between the focal lesion and the surrounding mucosa. Endoscopic imaging at the cancer invasion site can detect the thickening and rigidity of the gastric walls as well as the swelling and the waffle-like appearance of the gastric folds, all of which are a result of extensive fibrosis. Making a definitive diagnosis is based on the detection of irregular erosions and small depressions and requires biopsy of the affected site(s). However, as cancer cells are often found in the submucosa or deeper layers, biopsy results are false negative in many cases. In such instances, deep tissue biopsy is necessary; however, there is currently no consensus on the ideal method for obtaining deep tissue biopsy material. In this report, we present the case of patient in whom endoscopic ultrasound-guided fine needle aspiration (EUS-FNA) was effective in achieving a histopathological diagnosis of advanced gastric LP. We also review the relevant medical literature.

## 2 Presenting concerns

A 46-year-old male with chief complaints of epigastric pain and poor appetite was initially evaluated by a physician at a local clinic. Esophagogastroduodenoscopy (EGD) revealed changes suggestive of advanced gastric LP. As biopsy findings revealed only changes associated with gastritis and the patient tested positive for *Helicobacter pylori*, he underwent a second endoscopic examination and repeat biopsy after the eradication. Results of the repeat biopsy were similar to those of the first biopsy. As a macroscopic examination led to the suspicion of advanced gastric LP, the patient was admitted to our institution for further evaluation.

## 3 Clinical findings

On admission, the patient's medical history was unremarkable, except for a history of allergies; his family history was not significant. The patient did not consume alcohol but was a 15-pack-year smoker. His height was 171.1 cm, and he weighed 63.2 kg; he had a body mass index of 21.6 kg/m^2^. His vital signs were within normal limits. On performing an examination, he was noted to have epigastric pain on pressure.

## 4 Diagnostic focus and assessment

His laboratory test results are presented in Table 1. Briefly, his hepatobiliary enzyme levels were not elevated; however, he was noted to have mild renal insufficiency and elevated platelet levels. The carcinoembryonic antigen level was within normal limits, whereas the carbohydrate antigen 19-9 level was elevated at 98 U/mL. The anti-*H pylori* antibody was positive at 30 U/mL.

**Table 1:**
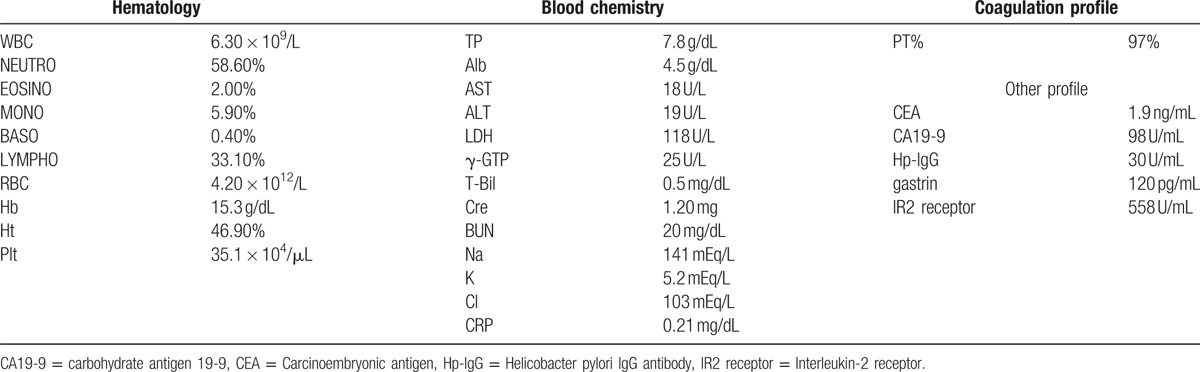
Summary of laboratory data.

**Figure 1:**
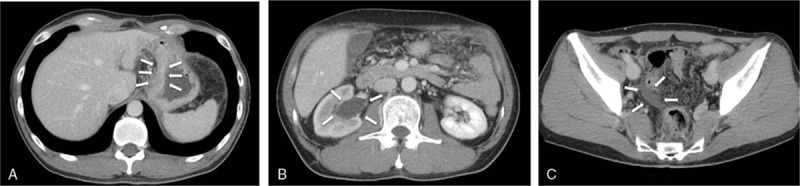
(A) Abdominal contrast-enhanced computed tomography (CT) suggested thickening of part of the posterior wall of the gastric corpus. (B) and (C). Right hydronephrosis and a small amount of ascites fluid were detected in the pelvic cavity.

**Figure 2:**
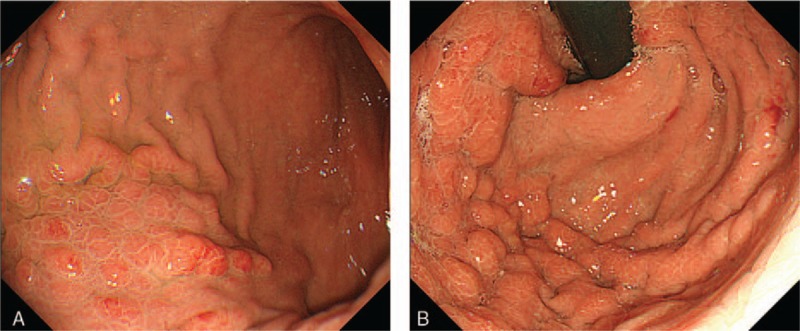
(A and B) Upper endoscopy revealed mucosal reddening and gastric fold swelling starting from the inferior portion of the greater curvature of the gastric corpus and extending to the fundus.

**Figure 3:**
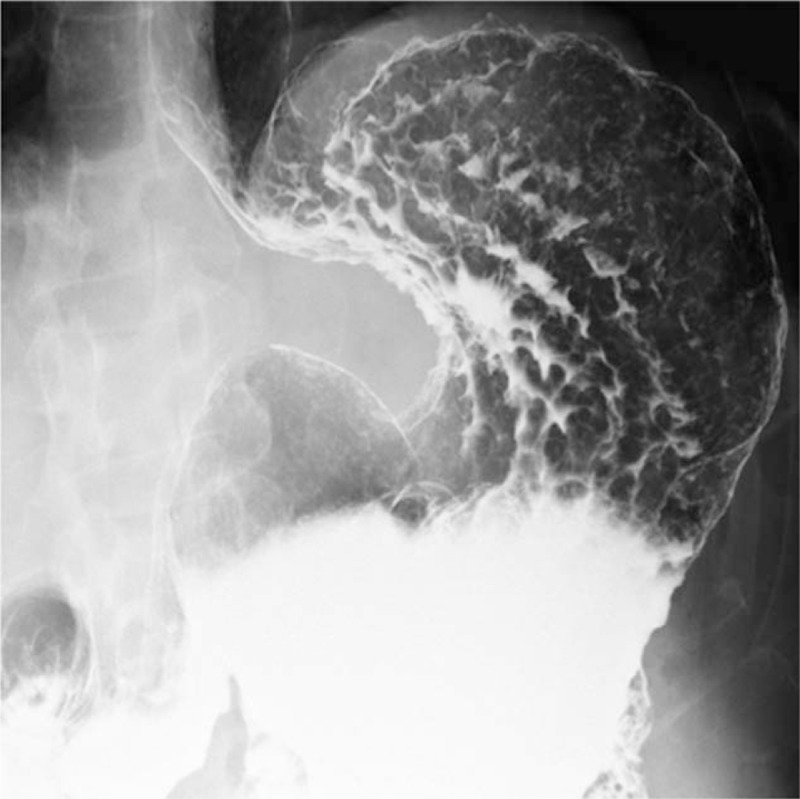
There were no irregular erosions or depressions within the field of view. Upper gastrointestinal series revealed gastric fold swelling extending from the gastric corpus to the fundus.

Abdominal contrast-enhanced computed tomography (CT) suggested thickening of part of the posterior wall of the gastric corpus (Fig. 1A–C). In addition, right hydronephrosis and a small amount of ascites fluid were detected in the pelvic cavity. No enlargement of associated lymph nodes was detected. Positron emission tomography (PET) did not indicate any abnormal accumulation of ^18^F-fluorodeoxyglucose, including accumulation in the stomach and the kidneys. Upper endoscopy revealed mucosal reddening and gastric fold swelling starting from the inferior portion of the greater curvature of the gastric corpus and extending to the fundus (Fig. 2A and B). No deformation or stricture of the pyloric antrum and canal was detected. There were no irregular erosions or depressions within the field of view. Upper gastrointestinal series revealed gastric fold swelling extending from the gastric corpus to the fundus (Fig. 3). While there were no strictures observed throughout the entire stomach, there was slight rigidity.

**Figure 4:**
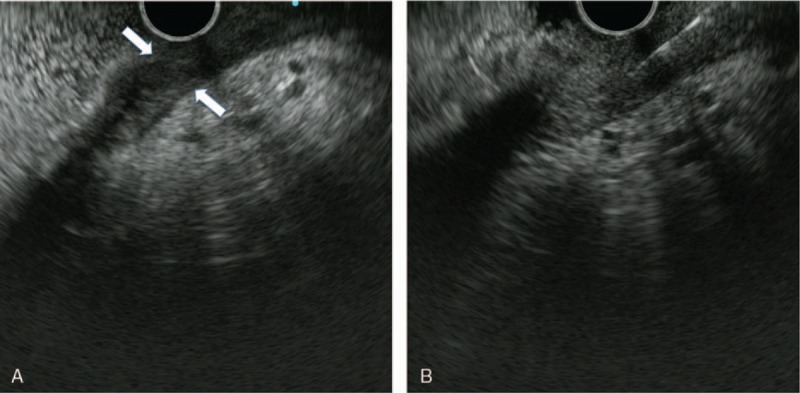
(A and B) Briefly, gastric wall thickening of up to 9.3 mm was observed mainly in the greater curvature. The layer structure was unclear, and slightly low-level echoes were detected in all layers. FNA was performed parallel to the gastric wall.

The patient underwent EUS-FNA (Fig. 4A and B). Briefly, gastric wall thickening of up to 9.3 mm was observed mainly in the greater curvature. The layer structure was unclear, and slightly low-level echoes were detected in all layers. FNA was performed parallel to the gastric wall using a 25G EchoTip Ultra; Cook Medical, Bloomington, IN and 10-cc syringe (10 strokes in three sections). As ascites was detected in the area surrounding the pancreas and on the inferior side of the liver, a sample of ascites fluid was collected by puncturing the stomach wall.

**Figure 5:**
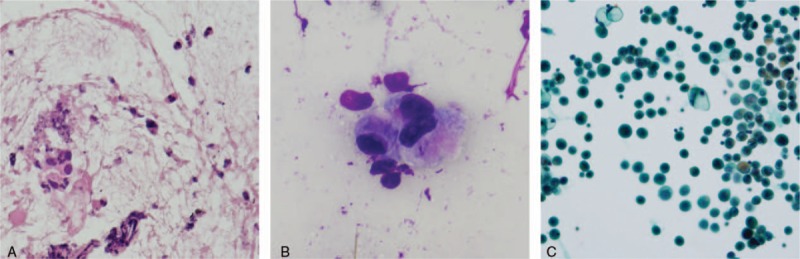
(A–C) Poorly differentiated adenocarcinoma cells were intermittently observed in sites other than the cellular cluster in the mucosa. Some of these scattered cancer cells showed mucus retention and uneven distribution of the nuclei. Papanicolaou staining of the ascites fluid showed cells with mucus retention. Based on the pathological findings in the gastric wall and the presence of ascites, the definitive diagnosis was signet ring cell carcinoma of the stomach.

Histopathological test results are presented in Fig. 5A–C. Poorly differentiated adenocarcinoma cells were intermittently observed in sites other than the cellular cluster in the mucosa. Some of these scattered cancer cells showed mucus retention and uneven distribution of the nuclei. Papanicolaou staining of the ascites fluid showed cells with mucus retention. Based on the pathological findings in the gastric wall and the presence of ascites, the definitive diagnosis was signet ring cell carcinoma (SRCC) of the stomach.

Abdominal CT which performed pre hospitalization indicated ascites and right ureteral obstruction, which led to the suspicion of peritoneal metastasis. In addition EGD which undergo after hospitalization indicated that the shape of the pyloric antrum was maintained, and advanced gastric LP was suspected. Normal and boring biopsies did not lead to a conclusive pathological diagnosis, and EUS-FNA was performed. Based on the pathological findings of the gastric wall and the presence of ascites, a definitive histopathological diagnosis of stage IV SRCC was made. A PET scan did not detect any abnormal accumulations suggestive of distal metastasis or the presence of a primary tumor; however, as there was low accumulation of fluorodeoxyglucose in PET in the SRCC, making a qualitative diagnosis was determined to be difficult.

## 5 Therapeutic focus and assessment, follow-up

In accordance with the patient's wishes, he was referred to another institution for chemotherapy.

## 6 Discussion

The incidence and mortality rates of gastric cancer, while declining globally, remain high in Asia and particularly in Japan. According to the International Agency for Research on Cancer (2008), Japan, South Korea, and China contained over 60% of gastric cancer patients.^[1]^ In Japan, while the risk of gastric cancer is higher in males, the rate of stage IV gastric cancer is particularly high among people younger than 40 years. Isobe et al^[1]^ reported that the frequency and 5-year survival rate of stage IV gastric cancer patients were 7% and 20.4%, respectively.

Gastric LP is an alternatively used definition for stage IV gastric cancer, and while both terms are widely considered synonymous, they exhibit distinctive characteristics based on the underlying pathological mechanisms of carcinogenesis. Stage IV gastric cancer evolves into a poor differentiated cancer from a differentiated cancer that arises from the atrophied mucosa of the antrum and inferior part of the gastric corpus. A mixed differentiated cancer proliferates and infiltrates to the submucosal layer. Stage IV gastric cancer is also characterized by a marked stricture of the pyloric antrum. In contrast, gastric LP is characterized by the presence of undifferentiated cancer cells in the mucosa of the fundic gland region and is a focal stage IIc intramucosal carcinoma. Diffuse infiltration into the submucosal tissue precedes ulceration of the stage IIc carcinoma and with the proliferation of fibrotic tissue is accompanied by rigidity emergence in the gastric wall, gastric wall thickening, and gastric fold swelling.^[2]^ As gastric LP is associated with early peritoneal and lymph node metastases, it has a poor prognosis. Pronounced gastric wall thickening can be identified by ultrasound and CT.^[3]^ An EUS-based diagnosis is made via the identification of the characteristic thickening of the third and fourth layers, which result from the submucosal or deeper infiltration of cancer cells and proliferation of fibrotic tissue. Once both fibrosis and cancer infiltration reach all layers, the 5-layer structure of the gastric wall can no longer be identified. Therefore, EUS allows the estimation of the invasion depth and extent of lymph node metastasis and aids in determining the TNM staging.^[4,5]^ However, it is difficult to accurately determine the extent of cancer cell infiltration using EUS alone as thickened areas caused by fibrosis may confound the findings. As the layer structure could not be identified by EUS in the present case, the walls were determined to have become fibrotic and the cancer was determined to have infiltrated all layers. However, the assessment of the extent of cancer infiltration was difficult.

It is critical to differentiate stage IV gastric cancer and advanced gastric LP from other diseases that might present with similar findings such as the gastric wall rigidity, gastric wall thickening, and gastric fold swelling. Some diseases that should be included in the differential diagnosis are malignant lymphoma, Ménétrier disease, gastric syphilis, gastroduodenal Crohn disease, corrosive gastritis, phlegmonous gastritis, gastric sarcoidosis, eosinophilic gastritis, and acute pancreatitis in pancreatic cancer.^[2,6]^ Endoscopic findings do not aid in the differential diagnosis in such cases. Instead, the clinical course and the patient's history at admission including details on diet, history of pharmaceutical use, and presence of malignancies and infectious diseases are important. Ultimately, making a pathological diagnosis using biopsy is necessary; however, a definitive diagnosis may not be made via normal biopsy.

As stage IV gastric cancer develops from differentiated cancer cells that are situated relatively close to the mucosal surface, normal biopsy is likely to lead to making a diagnosis. In contrast, making a definitive diagnosis of advanced gastric LP requires the identification of the depressed gastric lesion, which is the primary focal lesion that should be biopsied. However, the identification of this lesion is difficult. As cancer cells are often in the submucosal or deeper layer, normal biopsy results will frequently be negative; in fact, it has been reported that in up to 30% of gastric LP cases, particularly those without mucosal lesions, a forceps tissue biopsy can be negative.^[7]^ In such cases, deep tissue biopsy should be considered. In addition to EUS-FNA, boring biopsy is another option. Other options include biopsy of the lesion after exposing deep layers via endoscopic mucosal resection with a cap (EMRC)^[8]^ or submucosal dissection^[9]^ and biopsy of the lesion after laparoscopically exposing the serosa.^[10]^ A search for studies on histopathological diagnosis via deep biopsy of advanced gastric LP in PubMed since 2000 determined that EUS-FNA, boring biopsy, boring biopsy after mucosal dissection, biopsy of the lesion after exposing deep layers via EMRC and submucosal dissection, and biopsy of the lesion after laparoscopically exposing the serosa were used in 2, 5, 19, 1, and 1 studies, respectively (Table 2). Zhou et al^[11]^ compared cases in which biopsy sites were identified via EUS and where either boring biopsy or boring biopsy after mucosal dissection was performed. They reported that the positive biopsy rate was 80.6% and that 19.4% of patients experienced bleeding as a complication. Another study reported a patient in whom boring biopsy following mucosal dissection did not detect cancer cells; a definitive diagnosis was subsequently made by surgery.^[12]^ Although our review of the literature identified only 2 patients in whom EUS-FNA was performed, neither patient experienced complications and diagnosis was made in both patients.^[13,14]^ However, there are few studies that used biopsy. Thus, the positive biopsy rate in stage IV gastric cancer remains unknown, with no current consensus on the robust biopsy method.

**Table 2:**
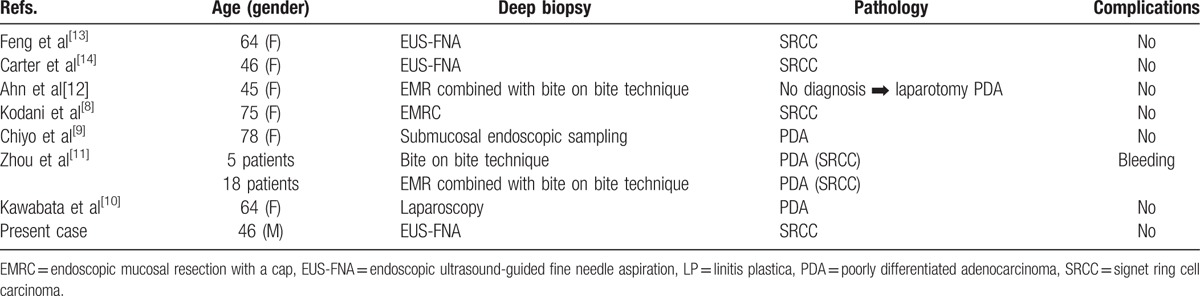
Diagnosis via deep biopsy.

In the present case, the previous physician performed boring biopsy based on clinical observations, which yielded a negative biopsy result. Similarly, at our institution, we were unable to locate an irregular erosion or small depression to suggest a primary focal tumor by performing EGD. Therefore, EUS-FNA was utilized for deep tissue biopsy, which led to making a diagnosis of gastric cancer.

Compared with biopsy after exposing deep layers, deep tissue biopsy using EUS-FNA allows for needle insertion after the confirmation of gastric thickening and local blood vessels, which markedly reduces the risk of bleeding and perforation. Therefore, this approach is a relatively noninvasive and effective method for tissue collection.^[15]^ In addition, in patients with small amounts of ascites fluid and in those with lymphadenopathy, EUS-FNA is an effective method for tumor staging. However, as advanced gastric LP presents as diffusely distributed cancer cells within fibrotic components, future studies should investigate the appropriate sites for needle insertion, the type and bore of the needle, and the number of strokes for obtaining the amount of tissue sample required for making a diagnosis.

Puncture needles used in EUS-FNA for making a histological diagnosis have been developed from cytological diagnostic tools. Making a cytological diagnosis alone is perceived to be insufficient by patients, and obtaining a sufficient amount of tissue sample to make a histological diagnosis by immunostaining and genetic screening has emerged as an important factor.^[16]^ Recently developed puncture needles have improved operability, even when large-bore needles, such as reverse-bevel side-port needles, Franseen needles, and 19 or 20 G needles, are used.^[17–19]^ Further case studies are needed to assess the effectiveness of EUS-FNA for gastric LP and to determine the types of needle and puncture techniques, positive biopsy rates, and procedural accidents. We expect that EUS-FNA can be utilized as a relatively noninvasive, highly sensitive, and specific pathological diagnostic procedure for advanced gastric LP.

### 6.1 Informed consent

I explained to the patient to announce this case report and confirmed that I got consent.
